# Affects of Anxiety and Depression on Health-Related Quality of Life among Patients with Benign Breast Lumps Diagnosed via Ultrasonography in China

**DOI:** 10.3390/ijerph120910587

**Published:** 2015-08-28

**Authors:** Zhe Lou, Yinyan Li, Yilong Yang, Lie Wang, Jun Yang

**Affiliations:** 1 Department of Cardiovascular Ultrasonic Diagnosis, the First Affiliated Hospital of China Medical University, No.155 Nanjingbei Road, Heping District, Shenyang 110001, China; E-Mail: blackpoppy@live.cn; 2 Department of Ultrasonic Diagnosis, The First Affiliated Hospital of China Medical University, No. 155 Nanjingbei Road, Heping District, Shenyang 110001, China; E-Mail: Eunyeonlee@gmail.com; 3 Department of Social Medicine, School of Public health, China Medical University, No.77 Puhe Road, Shenyang North New Area, Shenyang 110013, China; E-Mails: yyylll390@163.com (Y.Y.); liewang@mail.cmu.edu.cn (L.W.)

**Keywords:** China, benign breast lumps, anxiety symptoms, depressive symptoms, health-related quality of life

## Abstract

There is a high incidence of benign breast lumps among women, and these lumps may lead to physical and psychological problems. This study aims to evaluate anxiety and depressive symptoms among patients with benign breast lumps diagnosed via ultrasonography and investigate their impacts on health-related quality of life (HRQOL). A cross-sectional survey was conducted in Shenyang, China, from January to November 2013. Data were collected with self-administered questionnaires, including the Zung Self-Rating Anxiety Scale (SAS), the Center for Epidemiologic Studies Depression Scale (CES-D), and the 36-item Short-Form Health Survey (SF-36), together with demographic characteristics, from patients of the Department of Breast Surgery of the First Affiliated Hospital of China Medical University. Hierarchical multiple regression analysis (HMR) was performed to explore the effects of anxiety and depression on HRQOL. The overall prevalences of anxiety (SAS score ≥ 40) and depression (CES-D scores ≥ 16) were 40.2% and 62.0%, respectively, and 37.5% of the participants had both of these psychological symptoms. The means and standard deviations of PCS and MCS were 75.42 (15.22) and 68.70 (17.71), respectively. Anxiety and depressive symptoms were significantly negatively associated with the HRQOL of patients with benign breast lumps diagnosed via ultrasonography. Women with benign breast lumps diagnosed via ultrasonography in China experienced relatively high levels of anxiety and depressive symptoms. Anxiety and depressive symptoms had significant negative impacts on both the mental and physical quality of life (QOL) of women with benign breast lumps. Beyond the necessary clinical treatment procedures, psychological guidance and detailed explanations of the disease should be offered to alleviate the anxiety and depressive symptoms and enhance the HRQOL of patients with benign breast lumps.

## 1. Introduction

Benign breast lumps, which are found in over 60% of patients referred to breast clinics [1,2], pose a great threat to women’s physical and psychological health. It has been reported that approximately one out of every two women develops some degree of fibrocystic breast lesion, and one out of every five women develops fibroadenoma [3]. In Shanghai, approximately one out of every 350 women is diagnosed with fibroadenoma before the age of 60 [4]. Although most benign lumps do not cause harm to the physical aspect of quality of life (QOL), which encompasses an individual's perceived level of physical, psychological, and social well-being [5], it has been reported that approximately one-third of women who undergo investigation and receive a benign diagnosis of their breast symptoms are unsure or not reassured about their breast health [6]. Some uncertainty may arise from the diagnostic efficacy of ultrasonography for benign breast lumps. Less than 10% of benign breast lumps are falsely diagnosed as breast cancer [2], and ultrasonography plays a major role in the identification and diagnosis of breast lesions. However, women with benign breast lumps are often quite concerned and anxious about their health condition because benign breast lumps are important risk factors for breast cancer [7]. In fact, many women remain anxious following a benign breast lump diagnosis [8,9]. Therefore, patients with benign breast lumps tend to develop psychological disorders following their diagnosis via ultrasonography.

Anxiety is one of the most common mental disorders in China and many other countries [10,11,12]. For women with benign breast lumps, being called back for further investigation after mammographic screening has been reported to be a stressful experience [13,14,15,16]. In most cases, ultrasonography takes the place of mammography for the initial diagnosis of breast disease in China. Furthermore, women diagnosed with benign breast lumps via ultrasound exam often have to undergo fine needle aspiration, which can also lead to increased levels of stress and anxiety [15,17]. It has also been found that anxiety disorders have a significant negative impact on work functioning [18].

Depression is exceedingly common in modern society and has become a major mental health issue worldwide. It has been estimated that depression will become the second leading cause of disability following ischemic heart disease by 2020 [19]. Many studies have found a high level of depressive symptoms among breast cancer patients [20,21,22,23]. With regard to benign breast diseases, Hughes *et al.* found that women diagnosed with benign breast disease had a high rate of depressive symptoms compared to women who participated in published surveys of the general population [24]. However, to date, far less attention has been paid to the depressive symptoms of benign breast lump patients, compared to breast cancer patients.

In summary, anxiety affects women’s QOL, and the negotiation of life tasks such as education, relationships, personal goals, and occupation [25], and depression can impair job performance and work productivity [26,27] as well as QOL [28]. Given the high incidence of benign breast lumps and the large population of China, the physical and psychological QOL of patients with benign breast lumps cannot be ignored and need to be evaluated and treated. The aims of the present study are to evaluate anxiety and depressive symptoms among patients with benign breast lumps diagnosed via ultrasonography and to investigate their impacts on women’s health-related quality of life (HRQOL).

## 2. Methods

### 2.1. Study Design and Sample

This was a cross-sectional study of anxiety and depressive symptoms and HRQOL in patients diagnosed with benign breast lumps via ultrasonography. All the patients in the present study were recruited from the Department of Breast Surgery of the First Affiliated Hospital of China Medical University located in Shenyang, the capital city of Liaoning Province, between January and November 2013. Patients were eligible to participate if they met the following criteria: Women diagnosed with benign breast lumps through ultrasonic examination in the Physical Examination Center; had no history of psychological disorders; were able to read, write, and agree to participate. Thus, all the patients had been referred to the ultrasonic examination of the breast either as part of a general health physical examination, or because they had felt a lump or other uncomfortable symptoms from the breast. Ultrasound results were presented to the patients in the form of paper reports. Approximately one week after they received the diagnosis from the ultrasonic examination, patients made an individual appointment with our Department of Breast Surgery. They agreed to undergo further exams (ultrasonography, mammography, fine needle aspiration cytology (FNAC) or surgical biopsy) and completed a self-administered questionnaire. A total of 397 patients were approached, and effective responses were obtained from 371 patients (effective response rate: 93.5%). The study received approval from the Committee on Human Experimentation of China Medical University. The procedure that was followed was in accordance with ethical standards, and all women provided written informed consent.

### 2.2. Measurement

#### 2.2.1. Demographic Characteristics

The demographic questionnaire obtained information on age, marital status, education, monthly income, sleep disturbance, chronic disease, and life events. “Marital status” was categorized as “single”, “married/cohabitating”, and “divorced/other”. “Education” was categorized as “junior college or lower”, “undergraduate”, and “graduate or higher”. “Monthly income” was categorized as “≤3000 RMB” and “>3000 RMB”. “Chronic disease” was defined as “yes” if a common chronic disease (e.g., hypertension, diabetes mellitus, coronary heart disease, chronic gastritis, cervical spondylosis, and neurodermatitis) was diagnosed.

#### 2.2.2. Anxiety Symptoms

Anxiety symptoms were measured with the 20-item Self-Rating Anxiety Scale (SAS) developed by Zung in 1971 [29]. A Chinese version of the SAS was used in the present survey. The scale has been proven to correspond well with the assessment of anxiety and has been found to be reliable in epidemiological surveys of the Chinese population [11,30]. Each item contains four options that describe how often the respondents had each feeling in the past week on a four-point Likert scale ranging from “none or a little of the time (less than one day)’’ (coded as 1) to ‘‘most or all of the time (five to seven days)’’ (coded as 4). The total score on the SAS was calculated, with higher scores indicating a higher degree of anxiety. The original author set the cutoff point raw score of 36 to define clinically significant anxiety symptoms [31]. However, according to previous studies of the Chinese population [11,30], we defined the anxiety group as those having a total raw score of above 40 in this study. For the anxiety group, we evaluated the extent of anxiety according to score intervals. Standardization was performed based on the SAS (raw data multiplied by 1.25); then, the SAS scores were classified by the following cutoff points: 50–59 was mild anxiety, 60–69 was moderate anxiety, and greater than 70 was severe anxiety [32]. The Cronbach’s alpha for SAS was 0.83 in our study.

#### 2.2.3. Depressive Symptoms

Depressive symptoms were measured with the 20-item Chinese version of the Center for Epidemiologic Studies Depression Scale (CES-D) [33]. Each item had four response categories that described how often the respondents had each feeling in the past week, ranging from “rarely or none of the time (less than one day)” (coded as 0) to “most or all of the time (five to seven days)” (coded as 3). The total score on the CES-D was calculated, with higher scores indicating a higher severity of depressive symptoms. Participants with CES-D scores ≥16 were classified into the depression group [34,35,36]. The CES-D has been widely used in the Chinese population and has shown good reliability and validity [37,38]. The Cronbach’s alpha was 0.876 in the present study.

#### 2.2.4. Health-Related Quality of Life

HRQOL was measured with the 36-item Short-Form Health Survey (SF-36). The SF-36 measures eight different concepts of health: physical functioning (PF), role limitations due to physical problems (RP), bodily pain (BP), general health (GH), vitality (VT), social functioning (SF), role limitations due to emotional problems (RE), and mental health (MH), which were selected from the Medical Outcomes Study inventory [39]. These eight dimensions can be aggregated into a physical component summary (PCS) and mental component summary (MCS). A score was calculated for each dimension and was transformed to obtain a value ranging from 0 to 100, with higher scores indicating better health [40]. The Mandarin version of the SF-36 is a valid and reliable tool for assessing HRQOL [41]. The Cronbach’s alpha for the PCS and MCS were 0.836 and 0.828, respectively, in this study.

### 2.3. Statistical Analysis

The data acquired in this study were analyzed by the SPSS v13.0 (SPSS Inc., Chicago, CA, USA) statistical program for Windows. Differences in categorical variables were examined by t-test and one-way ANOVA. Pearson’s correlation was performed to test the correlation among continuous variables. All statistical tests were two-sided (α = 0.05). With QOL scores (PCS and MCS) as dependent variables, hierarchical multiple regression (HMR) analysis was performed to test the incremental variance by a given set of independent variables. In the present study, participants’ characteristics were entered into the first step of the regression model, depressive symptoms were entered into the second step, and anxiety symptoms were entered into the third step. The relative importance of the variables retained in the final multiple regression models contributed to the explained variance of QOL, which was represented as the standardized β [42]. Adjusted R^2^-value was used to assess the fit of the model. Prior to the regression analysis, all study variables were standardized to avoid multicollinearity. Additionally, we checked for multicollinearity using tolerance and variance inflation factor.

## 3. Results

### 3.1. Description of Participants and Distribution of Study Variables

Effective responses were obtained from 371 eligible women diagnosed with benign breast lumps through B-ultrasonic examination. Among them, 286 (77.1%) women were diagnosed with nodularity, which was the most common cause of benign breast lumps. A total of 31 (8.4%) women were diagnosed with fibroadenoma, and 39 (10.5%) women were diagnosed with cystic nodule. The proportions of cyst, intraductal papilloma, and mammary duct ectasia diagnoses were 1.1% (*n* = 4), 1.1% (*n* = 4), and 1.9% (*n* = 7), respectively.

Demographic characteristics and distributions of categorical study variables are presented in Table 1. In the present study, the mean and standard deviation of the SAS and CES-D scores were 37.50 (7.45) and 17.88 (7.88), respectively. The overall prevalence of anxiety (SAS score ≥ 40) and depressive (CES-D scores ≥ 16) symptoms were 40.2% and 62.0%, respectively, and 37.5% of the participants had both of these psychological symptoms. In total, 107 (28.8%) patients in our study were classified as having mild anxiety; 40 (10.8%) subjects as having moderate anxiety; and only 2 (0.5%) participants as having severe anxiety. The means and standard deviations of PCS and MCS were 75.42 (15.22) and 68.70 (17.71), respectively. Mental QOL was significantly lower than physical QOL (*p* = 0.000). The mean (SD) age of participants at the time of the interview was 40.31 (6.91) years, ranging from 24 to 60 years (Table 2).

Participants who were married/cohabitating had a significantly lower level of depressive symptoms than those who were single or divorced/other (*p* = 0.044). Those who had sleep disturbances showed significantly higher levels of anxiety (*p* = 0.000) and depressive symptoms (*p* = 0.000) and lower levels of physical (*p* = 0.012) and mental (*p* = 0.000) QOL. Regarding chronic disease, participants suffering from chronic diseases had significantly higher levels of anxiety (*p* = 0.002) and depressive (*p* = 0.045) symptoms and lower levels of physical (*p* = 0.007) and mental (*p* = 0.028) QOL than those who did not have a chronic disease.

**Table 1:** Demographic characteristics of participants, means and standard deviations (SDs) of variables.

Variable	N (%)	Anxiety	Depression	PCS	MCS
		Mean (SD)	Mean (SD)	Mean (SD)	Mean (SD)
Total	371	37.50 (7.45)	17.88 (7.88)	75.42 (15.22)	68.70 (17.71)
Age					
≤30	29 (7.8)	35.03 (6.48)	18.38 (8.72)	75.31 (15.53)	65.36 (19.01)
31–40	156 (42.0 )	37.79 (7.19)	17.79 (7.66)	74.78 (15.17)	67.38 (17.85)
41–50	167 (45.0)	37.44 (7.67)	17.66 (7.97)	76.15 (14.63)	70.24 (17.33)
>50	19 (5.1)	39.37 (8.62)	19.74 (7.98)	74.41 (20.56)	71.02 (17.64)
Marital status					
Single	27 (7.3)	36.74 (7.20)	20.44 (9.08)	71.09 (17.76)	65.32(19.84)
Married/cohabitating	331 (89.2)	37.51 (7.45)	17.53 (7.73) *	75.74 (15.01)	68.96(17.58)
Divorced/other	13 (3.5)	38.69 (8.36)	21.46 (7.84)	76.17 (14.95)	68.86(17.23)
Education					
Junior college or lower	140 (37.7)	37.88 (7.20)	18.19 (7.99)	76.21 (13.90)	67.97 (17.94)
Undergraduate	185 (49.9)	37.49 (7.61)	18.11 (7.63)	74.09 (16.27)	69.13 (17.50)
Graduate or higher	46 (12.4)	36.39 (7.63)	16.00 (8.46)	78.36 (14.45)	69.16 (18.20)
Monthly income					
≤3000	113 (30.5)	38.42 (7.35)	18.59 (8.50)	74.41 (14.55)	67.33 (17.24)
>3000	258 (69.5)	37.10 (7.48)	17.56 (7.59)	75.86 (15.52)	69.29 (17.91)
Sleep disturbance					
No	187 (50.4)	35.70 (7.08) ***	16.04 (7.89) ***	77.73 (14.53) *	72.26 (17.49) ***
Moderate	159 (42.9)	39.16 (7.25)	19.56 (7.30)	73.17 (15.50)	64.75 (17.11)
Severe	25 (6.7)	40.44 (8.27)	20.88 (8.37)	72.40 (16.46)	67.10 (18.20)
Chronic disease					
Yes	201(54.2)	38.69(7.33) **	18.69 (7.84) *	73.35 (15.90) **	66.78 (17.26) *
No	166(44.7)	36.23(7.36)	17.03 (7.88)	77.66 (14.14)	70.86 (18.21)

PCS, physical component summary; MCS, mental component summary; * *p* < 0.05, ** *p* < 0.01, *** *p* < 0.01(two-tailed).

**Table 2:** Means, standard deviations (SDs) and correlations of continuous variables.

Variable	Mean	SD	1	2	3	4
1. Age	40.31	6.91				
2. Anxiety	37.50	7.45	0.066			
3. Depression	17.88	7.88	−0.018	0.721 **		
4. PCS	75.42	15.22	0.026	−0.428 **	−0.363 **	
5. MCS	68.70	17.71	0.112 *	−0.470 **	−0.508 **	0.675 **

PCS, physical component summary; MCS, mental component summary; * *p* < 0.05, ** *p* < 0.01 (two-tailed).

### 3.2. Correlation between Study Variables and Effects of Anxiety and Depressive Symptoms on HRQOL

The results of Pearson’s correlation are presented in Table 2. Age was significantly correlated with mental QOL (*r* = 0.112, *p* = 0.031). Anxiety symptoms were positively correlated with depressive symptoms (*r* = 0.721, *p* = 0.000) and negatively correlated with PCS (*r* = −0.428, *p* = 0.000) and MCS (*r* = −0.470, *p* = 0.000). Furthermore, depressive symptoms were negatively correlated with PCS (*r* = −0.363, *p* = 0.000) and MCS (*r* = −0.508, *p* = 0.000).

HMR was performed to examine whether the anxiety and depressive symptoms had significant adverse effects on physical QOL (Table 3) and mental QOL (Table 4). After controlling for some demographic characteristics (age, marital status, education, income, sleep disturbance, life events, and chronic disease), anxiety and depressive symptoms were significantly negatively related to PCS and MCS. In the regression model, anxiety and depressive symptoms explained 15.4% of the variance in PCS and 23.3% of the variance in MCS. More specifically, anxiety symptoms explained 5.6% and 2.2% of the variance in PCS and MCS, respectively, and depressive symptoms explained 9.8% and 21.1% of the variance in PCS and MCS, respectively.

**Table 3:** Hierarchical multiple regression predicting PCS scores.

Variable	Options	PCS		
		Step1 (β)	Step2 (β)	Step3 (β)
Demographic characteristics				
Age		−0.002	−0.001	0.027
Marital status				
	(Single *vs.* Married/cohabitating)	−0.085	−0.054	−0.083
	(Divorced/other *vs.* Married/cohabitating)	0.021	0.043	0.024
Educational level				
	(Junior college or lower *vs.* Graduate or higher)	−0.040	−0.032	−0.051
	(Undergraduate *vs.* Graduate or higher)	−0.108	−0.093	−0.107
Income		0.042	−0.004	−0.036
Sleep disturbance				
	(Moderate *vs.* No)	−0.117 *	−0.049	−0.020
	(Severe *vs.* No)	−0.072	−0.025	−0.007
Life events	(Yes *vs.* No)	−0.066	−0.074	−0.088
Chronic disease	(Yes *vs.* No )	−0.134 *	−0.099 *	−0.068
Depressive symptoms			0.331 ***	−0.085
Anxiety symptoms				−0.354 ***
Adjusted R^2^		0.034 *	0.133 ***	0.189 ***
ΔR^2^		0.061	0.098	0.056

PCS: physical component summary; * *p* < 0.05, ** *p* < 0.01, *** *p* < 0.001.

**Table 4:** Hierarchical multiple regression predicting MCS scores.

Variable	Options	MCS		
		Step1 (β)	Step2 (β)	Step3 (β)
Demographic characteristics				
Age		0.122 *	0.123 *	0.140 **
Marital status				
	(Single *vs.* Married/cohabitating)	−0.036	0.009	−0.009
	(Divorced/other *vs.* Married/cohabitating)	0.006	0.039	0.027
Educational level				
	(Junior college or lower *vs.* Graduate or higher)	−0.018	−0.005	−0.018
	(Undergraduate *vs.* Graduate or higher)	0.040	0.061	0.052
Income		0.051	−0.017	−0.037
Sleep disturbance				
	(Moderate *vs.* No)	−0.196 ***	−0.096 *	−0.078
	(Severe *vs.* No)	−0.081	−0.012	0.000
Life events	(Yes *vs.* No)	−0.062	−0.074	−0.083
Chronic disease	(Yes *vs.* No )	−0.108 *	−0.057	−0.037
Depressive symptoms			0.485 ***	0.330 ***
Anxiety symptoms				−0.224 **
Adjusted R^2^		0.056 **	0.271 ***	0.292 ***
ΔR^2^		0.082	0.211	0.022

MCS: mental component summary; * *p* < 0.05, ** *p* < 0.01, *** *p* < 0.001.

## 4. Discussion

In the present study, we evaluated the presence of anxiety and depressive symptoms among patients who received a diagnosis of benign breast lumps via ultrasonography, and the impacts of these symptoms on women’s HRQOL. The prevalence of anxiety (SAS score ≥ 40) and depressive symptoms (CESD score ≥ 16) was 40.2% and 62.0%, respectively, and 37.5% participants had both of these psychological symptoms. Anxiety and depressive symptoms were significantly negatively associated with the women’s HRQOL.

The prevalence of anxiety symptoms among patients with benign breast lumps diagnosed sonographically found in this study is considerably higher than the 5.0%–6.3% one-month prevalence of anxiety disorder in China [12] and the reported lifetime rate of 14.6% in the United States [43]. According to Chinese studies that used the SAS to assess anxiety symptoms with a raw score of 40 as the cutoff point, our result is also higher than that of other populations such as Chinese medical students (12.5%) [30], middle and elementary school teachers (5.19%) [44], and even cancer patients (32.7%) in a district of Shanghai [45]. Regarding depression, judged according to the same criterion, our result is higher than the prevalence of 33.7% found in the general population in Chinese urban areas [46], the prevalence of 41.6% found in Chinese civil servants [47], and the prevalences in the general population in Japan (25.9% for males and 30.1% for females) [48] and Korea (26.1% for males and 28.7% for females) [49]. These findings reflect previous studies’ results that patients with benign breast disease tends to have more feelings of unhappiness and discontent [50] and psychological disorders [24,51,52].

Such astounding prevalences of anxiety and depression may be due to the location of the lumps. The breast is an organ unique to women. A previous survey on the QOL of women with breast cancer [53,54] revealed that the conservation and reconstruction of the breast can give women a new sense of control over their treatment and are quite successful in helping women feel comfortable with their bodies again. The breast is not only a biological organ; it also represents femininity, sexuality, and dignity.

The second explanation for these findings is the uncertainty of the diagnostic efficacy of ultrasonography for benign breast lumps. Benign breast lesions form an extremely heterogeneous group [55]. Some lesions are easily diagnosed due to their characteristic appearance; others are difficult to differentiate from malignant lesions. A standard examination carried out by an experienced sonographer using equipment with the latest technology has shown an elevated degree of sensitivity and specificity due to the possibility of compound and harmonic imaging, which improves the visibility of the margins and the echostructure of the lesion [56]. In our study, ultrasound examination was performed in the standard mode in the Physical Examination Center, without novel techniques such as the Automated Breast Volume Scanner (ABVS) or elastography; thus, specificity and sensitivity may be subtly influenced. According to recent studies, the diagnostic performance of B-mode ultrasound alone for the differentiation of benign and malignant breast lesions was good, with an area under the receiver operating characteristic of 0.851 [57]. Another study revealed that the specificity, sensitivity, and accuracy of the diagnosis of malignant breast tumors using conventional ultrasound were 80.0%, 81.1%, and 81.7%, respectively [58]. The uncertainty mentioned earlier usually led to further examinations through contrast-enhanced ultrasonography or fine needle aspiration cytology. Both of these techniques have a high specificity in diagnosing malignant breast lesions, which is approximately equal to significant sensitivity in the diagnosis of benign breast lumps [59]. These invasive examinations might irritate the patient and likely result in higher levels of stress and anxiety [13,14,15,25]. It is clear that the risk of the benign breast lumps becoming cancerous could be the source of anxiety and depression. Our findings implied that patients with benign breast lumps diagnosed sonographically in China experienced relatively high levels of anxiety and depressive symptoms. However, it must be noted that we only measured the psychological symptoms approximately one week after the ultrasonic examination. Because the status of patients’ anxiety and depressive symptoms is not constant [21,60,61,62], further research is needed to evaluate the level of these symptoms over different time periods following diagnosis.

It is not uncommon for a physical illness to trigger anxiety, but untreated and prolonged anxiety may aggravate physical symptoms, disturb patients’ QOL and increase side effects [63]. Anxiety affects women’s QOL and the negotiation of life tasks such as education, relationships, personal goals, and occupation [25]. Anxiety as an illness could markedly compromise individuals’ QOL and psychosocial functioning [43]. Significant impairment can even be found in individuals with sub-threshold forms of anxiety disorders [43]. Depression, a state of low mood and aversion to activity, can also impair QOL [28], and it even implies a persistent impairment of social functioning and living conditions [64]. For patients with advanced cancer, anxiety and depression were associated with impaired QOL, and they independently contributed to various dimensions of QOL such as global health status, emotional and cognitive functioning, and fatigue [65]. Similarly, for patients with chronic diseases such as diabetes mellitus, anxiety and depression were important factors associated with QOL [66].

Consistently, in the present study, we found that anxiety and depressive symptoms were significantly negatively related to the HRQOL of patients with benign breast lumps diagnosed via ultrasonography. Overall, anxiety and depressive symptoms explained 15.4% of the variance in physical QOL and 23.3% of the variance in mental QOL. Anxiety symptoms explained 5.6% and 2.2% of the variance in PCS and MCS, respectively, and depressive symptoms explained 9.8% and 21.1% of the variance in PCS and MCS, respectively. As evident, compared with anxiety, depression explained much more of the variance in MCS and PCS. Thus, depression was likely to cause more impairment to the physical and mental health of women with benign breast lumps diagnosed via ultrasound exam than anxiety. Some investigators support a chicken-or-egg theory of anxiety and depression [67]; however, in our cohort, depression showed a higher status than anxiety.

Our results indicated that anxiety and depressive symptoms both contributed to the impairment of HRQOL in patients with benign breast lumps diagnosed via ultrasonography in China; this finding is similar to that found in patients with cancer or other chronic diseases. Therefore, in addition to necessary clinical treatment procedures, psychological guidance should be offered to patients with benign breast lumps by the sonographer during the examination to adjust patients’ psychological state and improve their subjective feelings and understanding of the disease in the diagnostic stage. In the therapeutic stage, a detailed explanation of the treatment plan and psychological guidance should be given to guarantee compliance and reliance on medical staff. Finally, in the phases of rehabilitation, psychological counseling from the doctor can alleviate hesitation to undergo sequential treatment and help the patient return to normal life. Thus, it is necessary for the doctor to explain both the physical and psychological aspects of benign breast lumps in detail to patients, which could improve patients’ understanding of the disease and avoid unnecessary psychological distress and uncertainty. With the alleviation of anxiety and depressive symptoms, the psychological condition of patients with benign breast lumps diagnosed sonographically will be improved and their HRQOL will be enhanced.

Moreover, we found that participants who were single or divorced/other had a higher level of depressive symptoms than those who were married. This result is consistent with previous research that showed that married individuals had a lower level of depressive symptoms and mental distress and a higher level of life satisfaction and subjective well-being [68]. In addition, those who had sleep disturbances or chronic diseases reported higher levels of anxiety and depressive symptoms. More attention should be paid to these subgroups of populations.

Several limitations of the present study must be mentioned. First, a cross-sectional design was employed in our study; therefore, the direction of causality of the associations among the variables cannot be established. Second, the psychological symptoms were collected at one point in time, which may lead to overestimation or under estimation of the level of psychological symptoms in the study population. Longitudinal data collection is needed in further studies. Finally, all the participants in the present study were recruited from the First Affiliated Hospital of China Medical University; therefore, our findings may not be sufficiently representative to be extended to patients from other districts of the country or other levels of hospitals.

## 5. Conclusions

Women with benign breast lumps diagnosed sonographically in China experience relatively high levels of anxiety and depressive symptoms. Anxiety and depressive symptoms have significant negative impacts on QOL and are considerable determinants of the HRQOL of patients with benign breast lumps. In addition to necessary clinical treatment procedures, psychological guidance and detailed explanations of the disease should be offered to patients with benign breast lumps diagnosed via sonography to adjust their psychological state and improve their subjective feelings and understanding of the disease. This measure would, in turn, alleviate their anxiety and depressive symptoms and enhance their HRQOL.
